# The Massachusetts Medical Society

**Published:** 1870-11

**Authors:** 


THE MASSACHUSETTS MEDICAL SOCIETY has struck out from its By-Laws the paragraph providing for the admission of medical graduates of Harvard University without examination by the censors; and has further resolved that "the action of the American Medical Association, in effect imposing conditions upon the rights of this Society, was ill considered and unwarranted;" and "that no delegates from the Society be sent to the next Annual Meeting of the American Medical Association."

